# Dietary *Pleurotus citrinopileatus* Polysaccharide Improves Growth Performance and Meat Quality Associated with Alterations of Gut Microbiota in Arbor Acre Broilers

**DOI:** 10.3390/foods13213426

**Published:** 2024-10-28

**Authors:** Nannan Zhou, Xiaoxiao Song, Changxi Wu, Shuangmin Liang, Liangyu Yang, Changrong Ge, Zhichao Xiao

**Affiliations:** 1 Livestock Product Processing and Engineering Technology Research Center of Yunnan Province, Yunnan Agricultural University, Kunming 650201, China; znn01042023@126.com (N.Z.);; 2 College of Food Science and Technology, Yunnan Agricultural University, Kunming 650201, China; 3 College of Veterinary Medicine, Yunnan Agricultural University, Kunming 650201, China

**Keywords:** *Pleurotus citrinopileatus* polysaccharide (PCP), meat quality, intestinal microbiota, metabolomics, high-throughput sequencing

## Abstract

Adding edible fungal polysaccharides to animal diets improves growth performance, meat quality, intestinal health, and immunity without adverse effects. This study aimed to evaluate the impact of *Pleurotus citrinopileatus* polysaccharide (PCP, including PCP250, PCP500, PCP750, and PCP1000 mg/kg) on the growth performance, meat quality, and microbial composition of Arbor Acre (AA) broilers (total 180) by metabolomics and high-throughput sequencing. The results showed that adding PCP enhanced chicken meat tenderness, redness (a*), and water retention and raised essential amino acids and flavor amino acids (such as umami and sweet amino acids) content. The metabolomics revealed that IMP, creatine, betaine, sarcosine, and taurine were related to improving meat quality in broilers by PCP addition. In addition, amino acid, purine, and lipid metabolism were the main metabolic pathways. Moreover, PCP could regulate muscle metabolism by increasing the relative abundance of *Lachnospiraceae* and *Lactobacillus* and the content of short-chain fatty acids (SCFAs). Therefore, PCP may become a promising new dietary supplement in the future, which may improve the yield and quality of broiler chickens.

## 1. Introduction

As China’s second largest meat product, chicken has balanced nutrition and a standard taste profile [1]. It is generally considered to be a dependable and superior source of animal protein due to its advantageous characteristics of being high in protein and low in fat [2]. In the past, adding antibiotics into feed to improve the performance of animals has been a common and long-standing process for the livestock and poultry industry. Although this process can enhance animal immunity and growth rate, it also affects the composition of intestinal microbes, and excessive or prolonged use can lead to the development of antimicrobial resistance genes in the gut microbiota and also damage the immune systems of livestock and poultry. Finally, antibiotic residues in food can result in serious food safety problems [3,4]. Furthermore, with the rapid development of China’s livestock and poultry breeding industry, the demand for dietary structure and animal food has increased significantly, while people are increasingly in favor of high-quality meat products. However, due to its large population and low land utilization, China will face the pressure of raw material shortage. Moreover, there is a weak production system for feed ingredients and a gap in the supply of feed ingredients in China, which limits the development of the farming industry. Therefore, alternative strategies must be developed.

Due to their significant efficiency and minimal toxicity, the biological activities of polysaccharides derived from edible fungi have garnered extensive research attention. Specifically, numerous studies have focused on the composition of edible fungi and the biological processes underpinning their function, such as immune regulation, antioxidation, and anti-aging [5]. Following the recent discovery of the biological activities of plant polysaccharides, their application in animal production has become more prominent. Studies have revealed that adding appropriate doses of plant polysaccharides in animal diets can improve animal performance and meat quality [6], regulate intestinal microbiota structures, and provide favorable conditions for microbial proliferation [7]. Wu et al. [8] showed that feeding *Astragalus* polysaccharide could improve the growth performance of chickens. Yang et al. [9] showed that adding *Polygonatum sibiricum* polysaccharide to broiler diets improved the broilers’ growth performance and intestinal structure. In addition, studies have found that the addition of edible fungi polysaccharides (from mushrooms) to feed can increase the number of beneficial bacteria (such as *Bifidobacteria*, *Lactobacilli*, etc.) and inhibit the proliferation of harmful bacteria (such as *Bacteroides*, *Escherichia coli*, etc.) in the intestine of chickens [10]. *Pleurotus citrinopileatus* is a wood rot edible fungus considered an important and precious economic fungus. Polysaccharides are one of the main active components in *Pleurotus citrinopileatus* and have many biological activities [11]. Much previous research has focused on the physiological functions of *Pleurotus citrinopileatus* polysaccharides, such as its antioxidant, anti-tumor, hypoglycemic, and hypolipidemic properties, and it has shown good results [11,12]. In light of this, PCP has been recognized as a potentially cost-effective alternative to antibiotics for improving broiler health.

Therefore, we hypothesized that the addition of PCP to broiler diets may improve the growth performance and meat quality characteristics of broilers and that its beneficial effects may alter the composition and diversity of intestinal microbes as well as promote an increase in beneficial microbes, thus facilitating the use of PCP as a feed additive in production practices. This study will provide a new perspective to further understand the potential mechanisms of feeding natural edible fungi polysaccharides on broiler growth performance and meat quality.

## 2. Materials and Methods

### 2.1. Preparing for PCP

A 60 g amount of the dry powder of *Pleurotus citrinopileatus* (purchased from Yunnan Dehua Fungi Industry Co., Ltd., Kunming, China) was weighed. The material–liquid ratio was 1:3 g/mL at a 60 °C extraction temperature. The solution was sonicated for 25 min and then extracted in a water bath for 150 min. The solution was then centrifuged twice at 6500 rpm for 25 min each time. The supernatant was combined by rotary evaporation and anhydrous ethanol in a ratio of 1:3 was added. The collected solution was placed in a refrigerator at 4 °C for 24 h. Following this, the collected solution was centrifugated at 6500 rpm for 25 min. The supernatant was poured out to obtain PCP. The extraction rate of the polysaccharides was 18.60%, and the content of polysaccharides was 56.64% by the phenol sulfuric acid method.

### 2.2. Experimental Design

A total of 180 one-day-old male AA broilers (BW: 42.73 ± 1.59 g, Hunan Shuncheng Industrial Co., Ltd., Changsha, China) were randomly separated into 6 groups (10 chicks per replicate, 3 replicates per group) for a 42-day feeding trial. Baseline diets (Table S1) were fed to the control group (CON), the antibiotic group (CTC) was fed a basal diet containing 50 mg/kg chlortetracycline, and the treated groups were fed basal diets including 250 (PCP250), 500 (PCP500), 750 (PCP750), and 1000 (PCP1000) mg/kg PCP (i.e., 250–1000 mg of PCP per kg of feed fodder added). All broilers were raised in iron mesh cages (1.2 m long, 0.8 m wide, and 0.4 m high). For days 1–7, the temperature of the coop was 33–35 °C and the humidity was 60–65%, after which the temperature was lowered weekly (1–2 °C) to 18–21 °C at 6 weeks of age and the humidity was gradually reduced to about 55%, and 12–14 h of light per day was provided. At the same time, each chicken was vaccinated regularly. An unrestricted supply of feed and water was made available to all broiler chickens throughout the test period.

### 2.3. Production Performance and Sample Collection

The daily feed intake was recorded and the body weight of each broiler was weighed weekly. The feed efficiency, including the average daily feed intake (ADFI), average daily gain (ADG), feed-to-gain ratio (F/G), and body weight (BW), was calculated on days 21 and 42. Daily mortality was also recorded.

In each replicate, two broilers with an age of 42 days were selected at random and weighed; the chicks were euthanized via electroshock (voltage: 30–50 V) and jugular bloodletting following a 12 h feed deprivation period. The breast muscle was dissected and part of it was used for an analysis of physical indicators, while another portion was rapidly frozen using liquid nitrogen and subsequently transported to the laboratory and stored at −80 °C. Cecal contents were placed in sterile containers and frozen in liquid nitrogen immediately before being stored at −80 °C.

### 2.4. Determination of Meat Quality

On day 42, breast meat samples (*n* = 6) were collected to immediately analyze pH, shear force, cooking loss, drip loss, and meat color. After slaughtering, the pH values (at 45 min and 24 h) of the breast muscle were determined using a pH meter (HANNAHI9025, Italy). Shear force measurements were conducted using a digital display muscle tenderness meter (C-LM3B, China). Chest muscles with intact fibers were taken from each group at a uniform size (5 × 1 × 1 cm). A colorimeter (CR-400 Chroma meter, Konica Minolta Sensing) was used to measure the color of the meat, outputting three metrics: lightness (L*), redness (a*), and yellowness (b*). Approximately 40 g of breast meat was collected from each sample in each group to measure the drip loss and cooking loss by a previous research method [13].

### 2.5. Composition of Free Amino Acids in Muscle

The measurement of free amino acid content referenced the method from Xiao et al. [14] with slight changes. A 40 mg sample of freeze-dried chicken breast was placed into a 20 mL ampoule bottle and 10 mL of a 6 M solution of hydrochloric acid was added. After ultrasonic vibration, the ampoule was sealed with a spray gun, placed in an oven, heated to 110 °C for 23 h, then taken out and cooled to 25 °C. After 0.3 mL of liquid was passed through filter paper, the filtrate was accumulated in a quartz crucible and evaporated to dryness using a water bath heated to 70 °C. Following this, 3 mL of sample diluent was added. The combination was sieved through a 0.22 μm organic microporous membrane, and 1 mL of the sample was analyzed using an amino acid automatic analyzer (SykamS433D/S433, Germany).

### 2.6. Untargeted Metabolomics Analysis

Subsequently, 20 mg of the sample was weighed and 1 mL of extraction solution (2:2:1 *v*/*v* mixture of methanol, acetonitrile, and water) was added, along with an isotope-labeled internal standard mixture. Samples were ground for 4 min and then sonicated in an ice-water bath (35 Hz, 5 min, repeated 3 times). After standing for 1 h (−40 °C), the supernatant was then collected after centrifugation for 15 min (12,000 rpm, 4 °C). Following this, the same amount of QC (Quality Control) sample plate test supernatant was mixed with each sample.

A Vanquish UHPLC system (Thermo Fisher Scientific, Waltham, MA, USA) was integrated with a Q Exactive HFX mass spectrometer (Thermo Fisher Scientific, Waltham, MA, USA) to perform the LC-MS/MS measurements. The system was integrated with an ACQUITY UPLC HSS T3 column (2.1 mm × 100 mm × 1.7 μm; Waters Corporation, Milford, MA, USA). For the elution gradient at a flow rate of 0.5 mL/min, a mobile phase mixture comprising 25 mM ammonium acetate and ammonia dissolved in water (A) and acetonitrile (B) was employed. The relevant parameters in the positive and negative ion modes are shown in Table S2.

### 2.7. 16S rRNA Sequencing and Analysis

Three cecal digesta samples were randomly selected from each group for the 16S rRNA sequencing. The E.Z.N.A.^®^ soil DNA Kit (Omega Bio-tek, Norcross, GA, USA) was used to extract bacterial DNA. Primers 338F (5′-ACTCCTACGGGAGGCAGCAG-3′) and 806R (5′-GGACTACHVGGGTWTCTAAT-3′) were employed to amplify the hypervariable region V3-V4 of the bacterial 16S rRNA gene by an ABI GeneAmp^®^ 9700 PCR thermocycler (ABI, Foster City, CA, USA). The purified amplicons were combined in equimolar proportions and subjected to paired-end sequencing on an Illumina MiSeq PE300 platform (San Diego City, CA, USA) using the standard protocols established by Majorbio Bio-Pharm Technology Co., Ltd. (Shanghai, China). The raw 16S rRNA gene sequencing reads were demultiplexed, quality-filtered by fastp version 0.20.2, and merged by FLASH version 1.2.7 based on previous research parameters [15]. Operational taxonomic units (OTUs) with a 97% similarity cutoff were clustered using UPARSE version 7.1.

### 2.8. Short-Chain Fatty Acid (SCFAs) Analysis

For short-chain fatty acid sample pretreatment, this study referred to the method as previously described [16]. The extract was amalgamated and filtered through a 0.22 μm organic microporous membrane before being evaluated on the computer. Instrument parameters are listed in Table S3.

### 2.9. Statistical Analysis

Statistical analysis was performed using SPSS (version 17.3, IBM, Amunk, NY, USA) based on the mean ± SD of the results. Comparisons between means were made using Duncan’s multiple range tests (one-way ANOVA), with *p* < 0.05 being considered significant. Metabolomics data were analyzed by using SIMICA 14.1 software. Correlation analysis was carried out utilizing the Ouyi cloud platform (https://cloud.oebiotech.cn/tool, accessed on March 6, 2023).

## 3. Results

### 3.1. Production Performance Analysis

Table 1 exhibits the effects of different doses of PCP on the growth performance of AA broilers. The PCP1000 group exhibited a higher ADFI, ADG, and BW during the first period (1 to 21 days) compared to the CON group (*p* < 0.05). Between the ages of 21 and 42 days, broilers from PCP500, PCP750, and PCP1000 showed a higher ADFI compared with the CON group (*p* < 0.05, Table 1). The ADG of PCP750 exhibited a significant increase of 24.90% (*p* < 0.05) compared to the CON group. Moreover, PCP1000 showed higher BW compared with the CON group (*p* < 0.05). In terms of the F/G, the PCP750 group was the lowest (*p* > 0.05). The above results showed that a high dose of PCP had a positive effect on the growth performance of broilers.

### 3.2. Breast Meat Quality and Muscle Composition

#### 3.2.1. Analysis of Basic Indicators

On day 42, no significant differences were observed in the pH between the groups at 45 min (pH_45min_) and 24 h (pH_24h_) after slaughter, and each group exhibited significantly lower values at 24 h than at 45 min (Table 2). Compared with the CON and CTC groups, the PCP groups showed a considerable decrease in shear force (*p* < 0.05, Table 2). Our results showed a variation in the cooking and dripping losses across groups, with PCP750 exhibiting significantly lower values (*p* < 0.05, Table 2), suggesting that the water retention of broiler breast muscle increased with the feeding of PCP. In addition, broilers in the PCP750 group showed higher a* values and lower b* values in breast muscle compared to the CON group (*p* < 0.05, Table 3). The PCP750 group showed lower b* values compared with the CON and CTC groups (*p* < 0.05, Table 3). These results indicated that dietary PCP supplementation can improve the tenderness and water retention of broiler muscles and improve meat color.

#### 3.2.2. Free Amino Acid Analysis

Amino acids play a critical role in the flavor of substances. Zhou et al. [17] divided free amino acids into four (umami, sweet, bitter, and tasteless amino acids) categories. In this study, the types and contents of free amino acids (FAAs) were similar among all groups (Table S4). The most abundant amino acids were glutamic acid (Glu), phenylalanine (Phe), leucine (Leu), aspartic acid (Asp), alanine (Ala), and ornithine (Orn). The results revealed that bitter, umami, and sweet amino acids were dominant in each group. For umami amino acids, the amount of Glu was increased by 81.39% (*p* < 0.05) and 59.43% (*p* < 0.05) in PCP750 compared to the CON and CTC groups, respectively. The total FAA content in PCP750 and PCP1000 was higher compared with the CON group, increasing by 47.94% (*p* < 0.05) and 53.18% (*p* < 0.05), respectively. Compared with the CON group, PCP increased the content of essential amino acids (Ala, Met, Tyr, and Leu) in chicken muscle, with the high-dose group demonstrating a superior effect, indicating that adding PCP to broiler diets can enhance the quality and enrich the nutritional content of meat.

### 3.3. Metabolomic Analysis

Untargeted metabolomics was utilized to screen differential metabolites to better understand chicken meat taste differences among groups. A total of 97 and 138 metabolites were detected in the breast meat of AA broilers in each group in ESI+ and ESI- modes, respectively (Tables S5 and S6), predominantly containing amino acids, fatty acids, alkaloids, vitamins, nucleosides, and their analogs, organic acids, etc.

#### 3.3.1. PCA and PLS-DA Analysis

**Figure 1:**
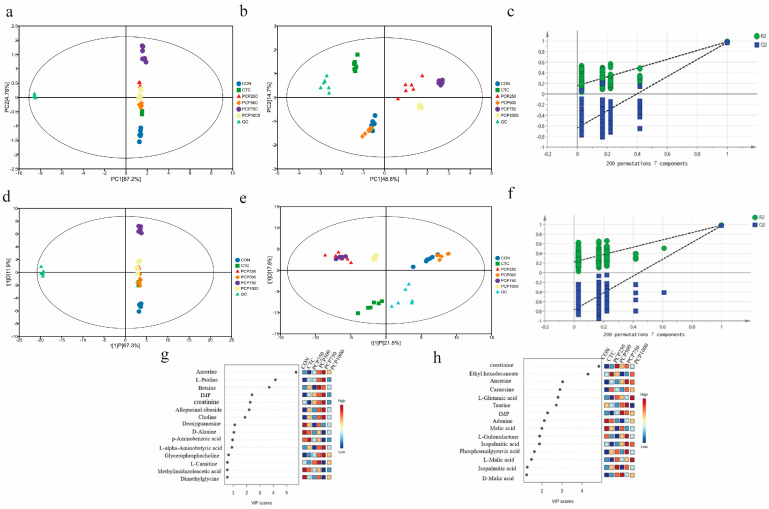
Metabolic profiles in breast muscle of each experimental group. PCA score plots and PLS−DA score plots of all groups in ESI+ (**a**,**d**) and ESI− mode (**b**,**e**), and permutations plots of PLS−DA models of ESI+ (**c**) and ESI− mode (**f**). VIP score chart in ESI+ (**g**) and ESI− mode (**h**).

The principal component analysis (PCA) charts of different groups in ESI+ and ESI− modes are shown in Figure 1a,b, revealing the reliability of the samples to fall within the 95% confidence interval. The sum of the two principal components was greater than 50% in both models, indicating that these two key components significantly contribute to elucidating the metabolite composition within the sample. These results indicated a clear differentiation pattern among the PCP, CON, and CTC groups, and differences in metabolite profiles of each group of samples.

The model was verified to be stable and dependable by a permutation test (200 permutations), with no excessive overfitting (Figure 1c,f). To comprehensively compare low molecular weight (LMW) compounds identified in the chicken breast meat of each group, the PLS-DA scores plot showed notable clustering and drift according to the addition of PCP (Figure 1d,e), suggesting that the metabolic profiles of the breast meat from each group differed. The cross-validated results showed that R^2^X (cum) = 0.96, R^2^Y (cum) = 0.985, and Q^2^ (cum) = 0.974 in ESI+ and R^2^X (cum) = 0.819, R^2^Y (cum) = 0.984, and Q^2^ (cum) = 0.969 in ESI-, implying that the model exhibits robust cumulative interpretation and predictive capabilities.

#### 3.3.2. Analysis of Differential Metabolites

Shown in Figure 1g (in ESI+) and Figure 1h (in ESI−) are the variable importance in the projection (VIP) plot values of muscle meat for all groups. An increased VIP value indicates a greater contribution of the corresponding metabolite [1]. In this study, 27 differential metabolites were identified (VIP > 1, *p* < 0.05) in the ESI+ and ESI− ion modes, of which carnosine, L-proline, betaine, inosine, creatinine, IMP, anserine, L-glutamic acid, taurine, etc., had VIP values significantly greater than 1, which were positively correlated with meat freshness indicators (Figure S1). These compounds are generally considered to be key biomarkers for assessing the quality of poultry meat. and have an impact on fresh meat quality, nutritional value, flavor, tenderness, juiciness, and functional differences [1,18].

#### 3.3.3. KEGG Pathway Analysis

**Figure 2:**
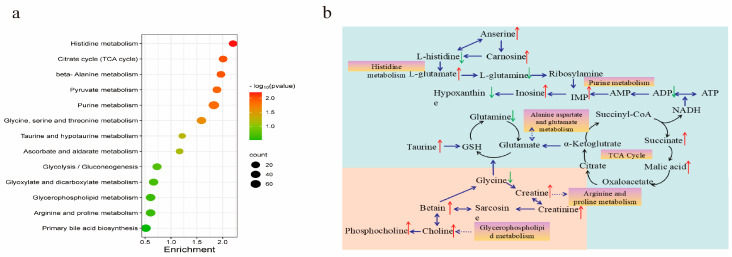
Metabolic pathway analysis. (**a**) Metabolic pathway enrichment analysis. (**b**) Key metabolic pathways contributing to meat quality between CON and PCP groups; red arrows represent up−regulation, and the green represents down−regulation of metabolites compared to the CON group, black arrows indicate the direction of metabolic pathways, and blue dotted arrows indicate the next metabolic pathway.

To explore the pathways of differential metabolites in the muscles of the PCP groups, KEGG enrichment analyses were performed for 27 important metabolites (Figure 2a). The results showed that thirteen metabolic pathways were significantly affected by PCP supplementation; among these pathways, histidine metabolism, citrate cycle (TCA cycle), beta-alanine metabolism, pyruvate metabolism, purine metabolism, and glycine, serine and threonine metabolism were the most enriched (*p* < 0.05). As shown in Figure 2b, based on the KEGG database, a pathway for the difference in metabolites of chicken breast muscle caused by the addition of PCP was identified. The key pathways were three amino acid metabolism pathways (histidine metabolism, arginine and proline metabolism, alanine aspartate and glutamate metabolism), one nucleotide metabolism pathway (purine metabolism), one lipid metabolism pathway (glycerophospholipid metabolism) and the TCA cycle, associating with the enrichment of anserine, taurine, betaine, creatinine, IMP, etc., in the muscle meat of the PCP groups. Furthermore, alterations in amino acid and purine metabolic pathways could potentially underlie the improved quality and composition of fresh meat after the incorporation of PCP, with the amino acid metabolic pathways specifically affirming an augmentation in the amino acid content within the breast meat.

### 3.4. Microbiota Diversity and SCFA Composition in the Caecum

#### 3.4.1. Analysis of the Microbial Composition of the Caecum

**Figure 3:**
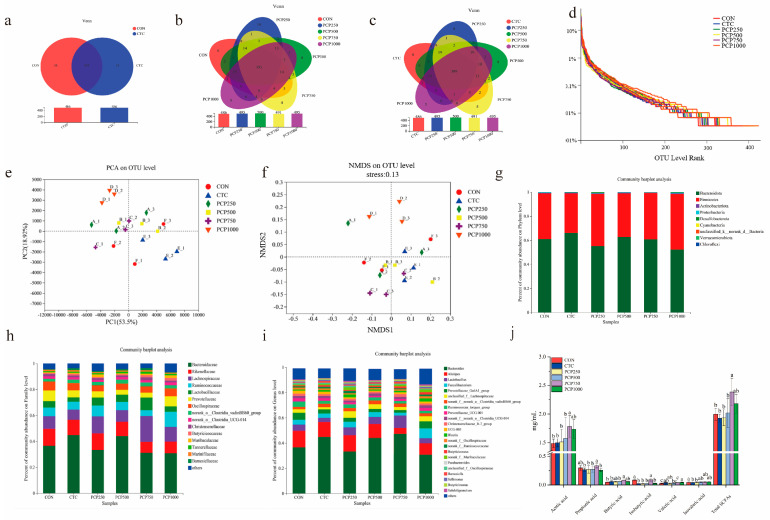
Effects of PCP on short−chain fatty acid (SCFAs) content and intestinal microbial community diversity and composition in the cecum of AA broilers. (**a**–**c**) Venn diagram analysis among groups: b represents the Venn diagram of caecal OTU number in the CON and CTC group; c represents the Venn diagram of caecal OTU number in the CON and PCP groups; d represents the Venn diagram of caecal OTU number in the CTC and PCP groups. (**d**) Rank-Abundance map in caecum microbiota of broilers. (**e**) PCA analysis of caecum microbiota of broilers. (**f**) NMDS analysis of caecum microbiota of broilers. (**g**–**i**) Relative abundance at the phylum (top 10), family (top 15), and genus (top 25) level in caecum microbiota of broilers. (**j**) Effect of different doses of PCP on the content of SCFAs of broilers. Means with different letters for each indicator are significantly different (*p* < 0.05).

To evaluate the impact of varying doses of PCP on the intestinal microflora, 16S rRNA sequencing was used to analyze the microbiota of broiler cecum. Figure 3a–c presents the Venn diagram for the number of OTUs in each group based on the species clustering results. At 42 days, 435 common OTUs were observed in the CON group and CTC, with 51 unique OTUs in each group (Figure 3a). There were 395 OTUs in common between the CON and PCP groups (PCP250, PCP500, PCP750, and PCP1000), with six, ten, eight, four, and five unique OTUs in each group (Figure 3b). There were 389 OTUs in common between the CTC and PCP groups, among which the number of OTUs specific to the groups were eight, eight, four, five, and five, respectively (Figure 3c). No significant difference was observed in terms of alpha diversity, including for the Abundance-based Coverage Estimator (ACE), Chao, and Coverage index (Figure S2a–e). The Shannon index was notably enhanced (*p* < 0.05), while the Simpson index exhibited a decline in the PCP1000 group compared with the CTC group, indicating that the PCP1000 group had higher richness and evenness. As shown in Figure 3d, the curves for each group were wide and flat, indicating that the species diversity of the samples in each group was high and relatively evenly distributed.

Subsequently, principal component analysis (PCA) and non-metric multidimensional scaling (NMDS) were used to compare the differences in intestinal microbiome samples in each group. The PCA results showed that the contribution of the first and second principal components was 53.50% and 18.92%, respectively. (Figure 3e), and the PCP-treated samples were distinct from those of the CON and CTC groups. The NMDS results also showed differences among the PCP, CON, and CTC groups (Figure 3f). These findings suggested that dietary PCP supplementation transformed the intestinal microbial composition and species distribution of the broilers.

#### 3.4.2. Microbial Community Structure Analysis

*Bacteroidota* and *Firmicutes* dominated the intestinal microbiota of broilers at the phylum level (Figure 3g). Among them, the relative abundance of *Bacteroidota* in the CTC was the highest, followed by the PCP500 group. The highest proportion of *Firmicutes* was observed in PCP1000, accounting for 46.12%, and the lowest was determined for CTC, accounting for 32.76%. At the family level, *Bacteroidaceae*, *Rikenellaceae*, and *Lachnospiraceae* were the dominant bacteria families in each group of samples (Figure 3h). Compared with the CON group, the relative abundance of *Lachnospiraceae* was increased, and the relative abundance of *Rikenellaceae* was decreased in the PCP750 group (*p* < 0.05). At the genus level, the dominant cecal intestinal flora in each group included *Bacteroides*, *Alistipes*, *Lactobacillus*, *Faecalibacterium*, and *Prevotellaceae_Ga6A1* (Figure 3i). In addition, the relative abundance of *Bacteroides* and *Lactobacillus* was the highest and *Alistipes* was the lowest in the PCP750 group.

#### 3.4.3. Short-Chain Fatty Acids (SCFAs) Analysis

As shown in Figure 3j, acetic acid, propionic acid, butyric acid, valeric acid, isovaleric acid, and total SCFAs presented a dose-dependent increase among the PCP250–PCP750 group compared to the CON group. The results of the network diagram analysis (Figure S2f) of the correlation between short-chain fatty acids and the dominant microbes in the intestine showed that the changes in the microbial composition of the cecum were closely related to SCFAs.

#### 3.4.4. Correlation Analysis Between Gut Microbiota and Meat Quality Index

**Figure 4:**
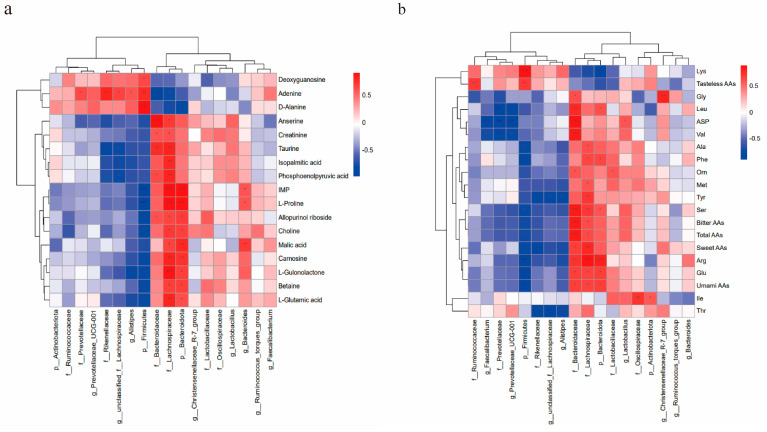
Heatmap analysis of Spearman’s correlation. (**a**) Heatmap of Spearman’s correlation between gut microbes and key differential metabolites. (**b**) Heatmap of Spearman’s correlation between gut microbes and flavor amino acids; significant correlation is marked by * *p* < 0.05, ** *p* < 0.01, and *** *p* < 0.001.

To further study the relationship between the changes in intestinal microbial composition and flavor amino acids and key metabolites, correlation analysis was carried out. A total of eight microbes were negatively correlated (*p* < 0.05) and ten were positively correlated (*p* < 0.05) with differential metabolites (Figure 4a) and amino acids (Figure 4b), respectively. Metabolites such as IMP, betaine, inosine, creatinine, anserine, and amino acids, which have an important effect on the flavor of meat products, showed a significant positive correlation with the increase of beneficial bacteria such as *Lachnospiraceae* and *Lactobacillus* as well as dominant bacteria such as *Bacteroidaceae* and *Bacteroides* in the PCP-treated groups. Interestingly, the key differential metabolites and amino acids in muscle were significantly negatively correlated (*p* < 0.05) with pathogenic bacteria such as *Alistipes* and *Rikenellaceae* in the PCP groups.

## 4. Discussion

Growth performance is an important indicator reflecting the growth and health of birds, the key content of bird individual identification, and the most representative indicator of individual quality [19]. In this study, ADFI, ADG, and BW were higher in the PCP750 group throughout the feeding phase, and the F/G was the lowest. Similar results were obtained when *Astragalus membranaceus* polysaccharide was added to the diet of juvenile broilers [8]. Previous research has indicated that diets containing *Glycyrrhiza* polysaccharide can enhance ADG and ADFI while reducing the F/G to varying degrees in broilers, and another study showed that the addition of *Radix rehmanniae praeparata* polysaccharide can increase body weight gain and reduce the F/G, which was consistent with our results [6,20]. In this study, our results suggested that a high level of supplementation of PCP can enhance the feed utilization efficiency and improve the growth performance of broilers. However, it is not always the case that a higher dose leads to a better effect. Consequently, the excessive addition of PCP in practical applications should be avoided, as a reasonable dosage will yield superior results.

Shear force is a valuable and intuitive indicator used to assess tenderness [21]. Prior research has reported that the addition of clove seeds to the diet is beneficial in terms of broilers’ meat tenderness [22], which concurred with our discoveries. What is more, this study indicated that PCP significantly reduced muscle cooking loss and dripping loss and effectively improved muscle water retention, which might be related to the pH_45min_ values of the groups. The lower the pH, the more lactic acid is produced by glycolysis, resulting in changes in the amount of net charge carried by proteins, which in turn affects the water retention of meat [23]. In addition, this also may be because PCP improves the composition of intestinal flora and enhances immunity and antioxidant capacity. These factors together lead to AA broilers to better maintain muscle water content and tenderness during growth, thereby improving the water-holding capacity of meat. Meat with a higher water retention capacity is reported to accelerate tenderization, thus improving meat quality [24,25]. Furthermore, PCP750 significantly increased muscle a* values in this study, which is associated with the antioxidant activity of PCP, while decreasing muscle b* values, and similar findings were gained by Wang et al. [25].

The amino acid composition of meat significantly influences both the nutritional quality of meat protein and its flavor, and the composition of necessary amino acids influences protein quality, whereas flavoring amino acids impact taste [26,27]. Free amino acids such as Glu, Asp, Gly, Lys, and Ala contribute to muscle flavor characteristics and have a great impact on meat flavor [13]. Such as lysine is exceedingly important in broiler nutrition and can promote its growth and development; aspartic acid can provide energy and participates in other amino acid metabolism [28]. Meanwhile, the addition of PCP can improve the concentration of essential amino acids in broiler muscle, thereby significantly contributing to the growth, immunity, and reproduction of broilers.

In this study, the PCP750 group exhibited the highest anserine, betaine, and IMP contents in both modes. Anserine is found mainly in the muscle tissue of most vertebrates. It can be used as an additional nutrient in meat due to its specific biological activities, such as specific antioxidant effects [29], suggesting that the elevated anserine levels in the PCP group could potentially augment the nutritional value of the poultry. Prior research has reported that dietary betaine can increase the contents of amino acids in breast muscle, reduce body fat deposition, improve the hydraulic and antioxidant capacity of the breast muscle system, and further improve the muscle quality of broilers [30]. In the present study, betaine content was significantly improved in the PCP500 and PCP750 groups, leading to the improved antioxidant capacity of the meat in the PCP supplementation group. Moreover, the umami flavor, an important meat flavor, mainly comes from ribonucleotide and umami amino acids [31]. IMP is decomposed from ribonucleotides, which can provide umami and increase sweetness at extremely low concentrations [32]. Additionally, IMP, carnosine, and anserine are essential precursors for forming flavor-related components in chicken meat [33]. Overall, the metabolomics results suggested that PCP can improve meat quality, nutritional value, functional properties, and broiler growth performance by up-regulating flavor-related metabolites such as carnosine, betaine, creatinine, anserine, IMP, etc.

Microbes in the cecum are highly complex and diverse and play a critical role in the intestinal health and growth of the host. Based on our results, dietary PCP had a selective effect on the gut microbiota, including up-regulating the abundance of beneficial bacteria such as *Lachnospiraceae* and *Lactobacillus*, and up-regulating the abundance of pathogenic bacteria such as *Alistipes* and *Rikenellaceae*. *Lachnospiraceae* can promote body health, including by providing nutrients to the host and providing energy to colonic epithelial cells, and can also maintain host immune homeostasis [34]. As an unclassified genus of *Lachnospiraceae*, the butyric acid-producing genus was positively correlated with the feed conversion and intestinal health of broilers [35]. *Lactobacillus* is essential for maintaining the equilibrium of gastrointestinal microecology in humans and animals. In this study, the relative abundance of *Lactobacillus* increased in the PCP750 groups and decreased in the CON group, which is consistent with the findings of De Cesare et al. [36]. Conversely, the family *Rikenbacteriaceae* has been shown to exacerbate pathogenicity through inflammation and is positively correlated with IL-1β, TGF-β1, and TNF-α levels [37]. The intestinal microflora is essential for preventing infectious diseases, regulating the digestion and metabolism of nutrients, and maintaining intestinal shape and immune homeostasis [15]. This study demonstrated that the addition of PCP improved the level of beneficial bacteria, resulting in enhanced intestinal development and improved nutrient utilization in broilers.

Changes in short-chain fatty acid production are a result of a variety of factors, including dietary habits, environment, disease, and medication. Numerous gut bacteria species can hydrolyze indigestible carbohydrates to produce short-chain fatty acids, primarily acetic, propionic, and butyric acids [38,39,40]. In this study, acetic, propionic, butyric, valeric, isovaleric, and isovaleric acids in PCP-treated cecum increased with the dose of PCP250–PCP750. The reason for the increase of SCFAs may be related to the fermentation process of PCP in the intestine. As a carbon source, polysaccharides are fermented by intestinal microorganisms to produce metabolites such as SCFAs, which not only provide an energy source for microorganisms but also have a positive impact on the physiological function and health status of broiler intestines. Previous studies have demonstrated the ability of SCFAs to stimulate enterocyte growth and proliferation [38]; therefore, one potential reason for improving gut health with PCP supplementation may be the increased levels of SCFAs in the gut. Additionally, it has been shown that *Firmicutes* is linked to the breakdown of polysaccharides and butyrate production, while *Bacteroidetes* are involved in the degradation of complex carbohydrates and the production of propionic acid through the succinic acid pathway [41]. Moreover, the anti-inflammatory qualities of butyric acid are useful in maintaining the integrity of the mucosal barrier [42]. Our study showed that PCP750 significantly increased the intestinal butyric acid content in broilers consistent with the higher relative abundance of the *Firmicutes*, suggesting that the intestines of broilers fed PCP may be healthier than those of the CON and CTC groups. However, this requires further histopathological analysis. Interestingly, the SCFA content of broilers in the CTC group decreased at 42 days of age in the present study; this may be attributed to the antibacterial effect of antibiotics, leading to the reduction of intestinal microbes. Studies have shown that a reduction in the concentration of SCFAs results in an elevation of the intraluminal pH level., which favors the growth of pathogenic or opportunistic pathogens, leaving the environment unrelated to the growth of beneficial bacteria [15]. In conclusion, dietary PCP may ultimately promote broiler growth by inducing the synthesis of SCFAs and thereby improving intestinal morphology, development, and health.

The correlation analysis showed that the high abundance of beneficial bacteria in the intestinal of broilers in the PCP groups was significantly positively correlated with key differential low molecular metabolites and flavor amino acids, indicating that the increase of intestinal beneficial bacteria had a positive effect on the meat quality and nutrition of broilers. This aligns with a prior investigation where an augmentation in the relative abundance of microorganisms exhibited a marked and positive correlation with elevated concentrations of flavor compounds, whereas a decline in certain genera was associated with a negative relationship [43]. These results indicated that changes in gut flora induced by PCP addition are closely related to the accumulation of amino acids and changes in the composition of different flavor metabolites in muscle.

Finally, the factors that inhibit the effects of high levels of PCP (PCP1000) addition on the regulation of meat quality in AA broilers were not known in this study. Although PCP has broad application prospects as a feed additive, further in-depth studies are imperative in elucidating the underlying mechanisms that govern its effects on meat quality regulation in AA broilers. This understanding is crucial if PCP is to be fully embraced and utilized as a feed ingredient by poultry producers globally.

## 5. Conclusions

This study demonstrated for the first time that PCP can be a beneficial and effective dietary supplement. Our study revealed that PCP750 significantly increased ADFI, ADG, and BW while decreasing the F/G in AA broilers. Fresh meat quality (pH, meat color, drip loss, etc.) was improved and the content of essential amino acids (Ala, Met, Tyr, and Leu) and the total amino acid content in broilers in muscle were notably enhanced in the PCP750 group. Amino acid metabolism, purine metabolism, and lipid metabolism were important metabolic pathways affecting broiler flavor. Moreover, PCP may promote intestinal health and the synthesis of SCFAs in the cecum of broilers by modulating the intestinal flora. Correlation analysis showed that the higher abundance of beneficial bacteria in the gut was significantly positively correlated with key meat quality indicators. The results of this study showed that the addition of PCP resulted in comparable or even better effects than antibiotics. These results could provide an effective natural feed additive capable of improving the growth performance and meat quality, offering a new approach to controlling the meat quality of broilers from farm to table.

## Figures and Tables

**Table 1:** The effect of PCP on growth performance of AA broilers.

	Group ^2^	CON	CTC	PCP250	PCP500	PCP750	PCP1000
Items ^1^							
1–21 days of age							
ADFI (g)		52.46 ± 0.69 ^c^	54.85 ± 0.96 ^ab^	53.07 ± 0.23 ^bc^	54.15 ± 0.99 ^abc^	56.22 ± 1.65 ^a^	55.22 ± 0.82 ^ab^
ADG (g)		38.51 ± 1.30 ^b^	39.96 ± 1.48 ^ab^	39.05 ± 1.79 ^ab^	40.41 ± 0.64 ^ab^	41.59 ± 0.72 ^a^	41.77 ± 0.73 ^a^
F/G		1.362 ± 0.029	1.372 ± 0.030	1.36 ± 0.058	1.34 ± 0.009	1.352 ± 0.019	1.323 ± 0.037
BW (g)		849.22 ± 27.14 ^b^	882.92 ± 31.24 ^ab^	860.93 ± 38.00 ^b^	891.70 ± 13.41 ^ab^	921.60 ± 16.30 ^a^	929.28 ± 10.65 ^a^
Mortality (%)		0.05	0	0	0	0	0
21–42 days of age							
ADFI (g)		137.74 ± 3.70 ^c^	159.01 ± 2.98 ^a^	137.91 ± 2.78 ^c^	149.00 ± 3.70 ^b^	164.29 ± 1.71 ^a^	152.22 ± 2.22 ^b^
ADG (g)		73.01 ± 5.91 ^c^	88.28 ± 2.02 ^a^	72.36 ± 0.56 ^c^	80.87 ± 3.66 ^b^	91.19 ± 2.85 ^a^	79.00 ± 1.50 ^bc^
F/G		1.895 ± 0.108	1.803 ± 0.071	1.906 ± 0.028	1.845 ± 0.075	1.802 ± 0.070	1.972 ± 0.034
BW (g)		2572.50 ± 92.34 ^b^	2775.67 ± 32.78 ^a^	2363.19 ± 100.23 ^c^	2678.08 ± 54.62 ^ab^	2838.67 ± 71.87 ^a^	2607.89 ± 63.26 ^b^
Mortality (%)		0.02	0	0	0.045	0	0.045

Means with different superscript letters in the same row are significantly different (*p* < 0.05, *n* = 6). ^1^ ADFI = average daily feed intake; ADG = average daily gain; F/G = feed/gain ratio; BW = body weight. ^2^ CON: control group, CTC: antibiotic group, and PCP: different doses of *Pleurotus citrinopileatus* polysaccharide treatment groups.

**Table 2:** Determination of pH, shear force, cooking loss, and drip loss in AA broiler’s breast meat.

	Items ^2^	pH_45min_	pH_24h_	Shear Force (kgf)	Cooking Loss (%)	Drip Loss (%)
Group ^1^						
CON		6.22 ± 0.193	5.76 ± 0.183	2.97 ± 0.316 ^a^	14.67 ± 1.198 ^b^	3.55 ± 0.238 ^b^
CTC		6.22 ± 0.089	5.76 ± 0.100	2.44 ± 0.273 ^b^	17.28 ± 1.716 ^a^	3.93 ± 0.351 ^a^
PCP250		6.29 ± 0.131	5.76 ± 0.093	2.44 ± 0.350 ^b^	18.98 ± 2.139 ^a^	2.47 ± 0.331 ^d^
PCP500		6.27 ± 0.160	5.80 ± 0.069	2.21 ± 0.429 ^b^	14.32 ± 1.500 ^b^	2.53 ± 0.253 ^d^
PCP750		6.31 ± 0.131	5.72 ± 0.117	2.08 ± 0.313 ^b^	11.82 ± 0.792 ^c^	2.42 ± 0.196 ^d^
PCP1000		6.20 ± 0.125	5.78 ± 0.100	2.31 ± 0.278 ^b^	14.13 ± 1.014 ^b^	2.95 ± 0.281 ^c^

Means with different superscript letters in the same row are significantly different (*p* < 0.05, *n* = 6). ^1^ CON: control group, CTC: antibiotic group, and PCP: different doses of *Pleurotus citrinopileatus* polysaccharide treatment groups. ^2^ pH_45min_ = 45 min post-slaughter; pH_24h_ = 24 h post-slaughter.

**Table 3:** Determination of meat color in AA broiler’s breast meat.

	Items ^2^	Meat Color		
Group ^1^		L*	a*	b*
CON		42.77 ± 1.579	4.67 ± 0.084 ^bc^	18.25 ± 2.518 ^a^
CTC		42.77 ± 1.457	3.61 ± 0.738 ^d^	17.31 ± 0.643 ^ab^
PCP250		44.19 ± 1.541	3.95 ± 0.349 ^cd^	16.90 ± 1.525 ^ab^
PCP500		43.87 ± 1.972	5.13 ± 0.701 ^ab^	15.69 ± 1.009 ^bc^
PCP750		41.60 ± 2.212	5.45 ± 0.723 ^a^	14.53 ± 1.306 ^c^
PCP1000		43.284 ± 2.112	4.38 ± 0.294 ^c^	16.99 ± 1.253 ^ab^

Means with different superscript letters in the same row are significantly different (*p* < 0.05, *n* = 6). ^1^ CON: control group, CTC: antibiotic group, and PCP: different doses of *Pleurotus citrinopileatus* polysaccharide treatment groups. ^2^ L*: luminance; a*: redness; b*: yellowness.

## Data Availability

The original contributions presented in the study are included in the article/Supplementary Material, further inquiries can be directed to the corresponding author.

## Supplementary Materials

The following supporting information can be downloaded at: https://www.mdpi.com/article/10.3390/foods13213426/s1, Table S1: Composition and nutrient level of the basic diet; Table S2: Detection parameters on the machine in positive and negative ion mode; Table S3: GC-MS online detection conditions; Table S4: The results of free amino acids composition and content in breast muscle of AA broilers (mg/g); Table S5: Metabolites in breast muscle of AA broilers in positive ion mode; Table S6: Metabolites in breast muscle of broilers in negative ion mode; Figure S1: Correlations between key meat parameters and differential metabolites of AA broilers. Figure S2. Analysis chart of influence of caecal alpha diversity in each group.

## References

[1] Xiao Z.C., Luo Y.T., Wang G.Y., Ge C.R., Zhou G.H., Zhang W.G., Liao G.Z. (2017). ^1^H-NMR-based water-soluble low molecular weight compound characterization and fatty acid composition of boiled Wuding chicken during processing. J. Sci. Food Agric..

[2] Fan M., Xiao Q., Xie J., Cheng J., Sun B., Du W., Wang Y., Wang T. (2018). Aroma Compounds in Chicken Broths of Beijing Youji and Commercial Broilers. J. Agric. Food Chem..

[3] Chen J., Ying G.G., Deng W.J. (2019). Antibiotic Residues in Food: Extraction, Analysis, and Human Health Concerns. J. Agric. Food Chem..

[4] Kim J., Ahn J. (2022). Emergence and spread of antibiotic-resistant foodborne pathogens from farm to table. Food Sci. Biotechnol..

[5] Sun Y., Zhang M., Fang Z. (2020). Efficient physical extraction of active constituents from edible fungi and their potential bioactivities: A review. Trends. Food. Sci. Technol..

[6] Yang B., Li X., Baran A.M., Abdel-Moneim A.E. (2023). Effects of dietary incorporation of *Radix rehmanniae praeparata* polysaccharide on growth performance, digestive physiology, blood metabolites, meat quality, and tibia characteristics in broiler chickens. Poultry Sci..

[7] Zhang S., Zhang M., Li W., Ma L., Liu X., Ding Q., Yu W., Yu T., Ding C., Liu W. (2023). Research progress of natural plant polysaccharides inhibiting inflammatory signaling pathways and regulating intestinal flora and metabolism to protect inflammatory bowel disease. Int. J. Biol. Macromol..

[8] Wu S. (2018). Effect of dietary Astragalus membranaceus polysaccharide on the growth performance and immunity of juvenile broilers. Poultry Sci..

[9] Yang B., Li X., Mesalam N., Elsadek M., Abdel-Moneim A. (2024). The impact of dietary supplementation of polysaccharide derived from polygonatum sibiricum on growth, antioxidant capacity, meat quality, digestive physiology, and gut microbiota in broiler chickens. Poultry Sci..

[10] Guo F., Williams B.A., Kwakkel R.P., Li H.S., Li X.P., Luo J.Y., Li W.K., Verstegen M.W.A. (2004). Effects of mushroom and herb polysaccharides, as alternatives for an antibiotic, on the cecal microbial ecosystem in broiler chickens. Poultry Sci..

[11] Pan K., Jiang Q., Liu G., Miao X., Zhong D.W. (2013). Optimization extraction of Ganoderma lucidum polysaccharides and its immunity and antioxidant activities. Int. J. Biol. Macromol..

[12] Wu D.-T., Meng L.-Z., Wang L.-Y., Lv G.-P., Cheong K.-L., Hu D.-J., Guan J., Zhao J., Li S.-P. (2014). Chain conformation and immunomodulatory activity of a hyperbranched polysaccharide from Cordyceps sinensis. Carbohyd Polym..

[13] Jiang X., Yang J., Zhou Z., Yu L., Yu L., He J., Zhu K., Luo Y., Wang H., Du X. (2023). Moringa oleifera leaf improves meat quality by modulating intestinal microbes in white feather broilers. Food Chem. X.

[14] Xiao X., Wang B.W., Zhao P., Ge C.R., Li S.J., Xiao Z.C. (2022). The effect of the improvement technology on the quality of Midu pork roll. Foods.

[15] Edgar R.C. (2013). Uparse: Highly accurate OTU sequences from microbial amplicon reads. Nat. Methods.

[16] Liu T., Tang J., Feng F. (2020). Glycerol monolaurate improves performance, intestinal development, and muscle amino acids in yellow-feathered broilers via manipulating gut microbiota. Appl. Microbiol. Biotechnol..

[17] Zhou C.Y., Wang Y., Cao J.X., Chen Y.J., Liu Y., Sun Y.Y., Pan D.D., Ou C.R. (2016). The effect of dry-cured salt contents on accumulation of non-volatile compounds during dry-cured goose processing. Poultry Sci..

[18] Ritota M., Casciani L., Failla S., Valentini M. (2012). HRMAS-NMR spectroscopy and multivariate analysis meat characterisation. Meat Sci..

[19] Abdollahi M.R., Zaefarian F., Ravindran V. (2018). Feed intake response of broilers: Impact of feed processing. Anim. Feed. Sci. Technol..

[20] Zhang C., Li C., Shao Q., Chen W., Ma L., Xu W., Li Y., Huang S., Ma Y. (2021). Effects of Glycyrrhiza polysaccharide in diet on growth performance, serum antioxidant capacity, and biochemistry of broilers. Poultry Sci..

[21] Bowker B.C., Eastridge J.S., Solomon M.B. (2011). Use of gelatin gels as a reference material for performance evaluation of meat shear force measurements. J. Food Sci..

[22] Suliman G.M., Alowaimer A.N., Al-Mufarrej S.I., Hussein E.O.S., Fazea E.H., Naiel M.A.E., Alhotan R.A., Swelum A.A. (2021). The effects of clove seed (*Syzygium aromaticum*) dietary administration on carcass characteristics, meat quality, and sensory attributes of broiler chickens. Poultry Sci..

[23] Young J.F., Bertram H.C., Rosenvold K., Lindahl G., Oksbjerg N. (2005). Dietary creatine monohydrate affects quality attributes of Duroc but not Landrace pork. Meat Sci..

[24] Qiao M., Fletcher D.L., Smith D.P., Northcutt J.K. (2001). The effect of broiler breast meat color on pH, moisture, water-holding capacity, and emulsification capacity. Poultry Sci..

[25] Wang Y., Zhou X., Liu M., Zang H., Zhang R., Yang H., Jin S., Qi X., Shan A., Feng X. (2023). Quality of chicken breast meat improved by dietary pterostilbene referring to up-regulated antioxidant capacity and enhanced protein structure. Food Chem..

[26] Yang F., Cho W.-Y., Seo H.G., Jeon B.-T., Kim J.-H., Kim Y.H.B., Wang Y., Lee C.-H. (2020). Effect of *L*-cysteine, *Boswellia serrata*, and whey protein on the antioxidant and physicochemical properties of pork patties. Foods.

[27] Wood J.D., Brown S.N., Nute G.R., Whittington F.M., Perry A.M., Johnson S.P., Enser M. (1996). Effects of breed, feed level and conditioning time on the tenderness of pork. Meat Sci..

[28] Zajíc T., Mráz J., Pickova J. (2016). Evaluation of the effect of dietary sesamin on white muscle lipid composition of common carp (*Cyprinus carpio* L.) juveniles. Aquac. Res..

[29] Sunagawa Y., Katayama A., Funamoto M., Shimizu K., Shimizu S., Sari N., Katanasaka Y., Miyazaki Y., Hosomi R., Hasegawa K. (2022). The polyunsaturated fatty acids, EPA and DHA, ameliorate myocardial infarction-induced heart failure by inhibiting p300-HAT activity in rats. J. Nutr. Biochem..

[30] Chen R., Yang M., Song Y., Wang R., Wen C., Liu Q., Zhou Y., Zhuang S. (2022). Effect of anhydrous betaine and hydrochloride betaine on growth performance, meat quality, postmortem glycolysis, and antioxidant capacity of broilers. Poultry Sci..

[31] Xiao Z.C., Ge C.R., Zhou G.H., Zhang W.G., Liao G.Z. (2018). 1H-NMR-based metabolic characterization of Chinese Wuding chicken meat. Food Chem..

[32] Johnson R.J., Nakagawa T., Sánchez-Lozada L.G., Lanaspa M.A., Tamura Y., Tanabe K., Ishimoto T., Thomas J., Inaba S., Kitagawa W. (2013). Umami: The taste that drives purine intake. J. Rheumatol..

[33] Liu X., Zhang Y.-R., Cai C., Ni X.-Q., Zhu Q., Ren J.-L., Chen Y., Zhang L.-S., Xue C.-D., Zhao J. (2019). Taurine alleviates schistosoma-induced liver injury by inhibiting the TXNIP/NLRP3 inflammasome signal pathway and pyroptosis. Infect. Immun..

[34] Shi S., Qi Z., Jiang W., Quan S., Sheng T., Tu J., Shao Y., Qi K. (2020). Effects of probiotics on cecal microbiome profile altered by duck Escherichia coli 17 infection in Cherry Valley ducks. Microb. Pathog..

[35] Torok V.A., Allison G.E., Percy N.J., Ophel-Keller K., Hughes R.J. (2011). Influence of antimicrobial feed additives on broiler commensal posthatch gut microbiota development and performance. Appl. Environ. Microbiol..

[36] De Cesare A., Sala C., Castellani G., Astolfi A., Indio V., Giardini A., Manfreda G. (2020). Effect of *Lactobacillus acidophilus* D2/CSL (CECT4529) supplementation in drinking water on chicken crop and caeca microbiome. PLoS ONE.

[37] Sun L., Zhang X., Zhang Y., Zheng K., Xiang Q., Chen N., Chen Z., Zhang N., Zhu J., He Q. (2019). Antibiotic-induced disruption of gut microbiota alters local metabolomes and immune responses. Front. Cell. Infect. Microbiol..

[38] Pan D., Yu Z. (2014). Intestinal microbiome of poultry and its interaction with host and diet. Gut Microbes.

[39] Flint H.J., Scott K.P., Louis P., Duncan S.H. (2012). The role of the gut microbiota in nutrition and health. Nat. Rev. Gastro. Hepat..

[40] Kim C.-S., Cha J., Sim M., Jung S., Chun W.Y., Baik H.W., Shin D.-M., Cha L. (2021). Probiotic supplementation improves cognitive function and mood with changes in gut microbiota in community-dwelling older adults: A randomized, double-blind, placebo-controlled, multicenter trial. J. Gerontol. A Biol. Sci. Med. Sci..

[41] Turnbaugh P.J., Ley R.E., Mahowald M.A., Magrini V., Mardis E.R., Gordon J.I. (2006). An obesity-associated gut microbiome with increased capacity for energy harvest. Nature.

[42] Yu M., Mu C., Zhang C., Yang Y., Su Y., Zhu W. (2018). Marked response in microbial community and metabolism in the ileum and cecum of suckling piglets after early antibiotics exposure. Front. Microbiol..

[43] Wang Y., Sun J., Zhong H., Li N., Xu H., Zhu Q., Liu Y. (2017). Effect of probiotics on the meat flavour and gut microbiota of chicken. Sci. Rep..

