# Supplementary material for: Dietary Pleurotus citrinopileatus Polysaccharide Improves Growth Performance and Meat Quality Associated with Alterations of Gut Microbiota in Arbor Acre Broilers
Source: Foods. 2024 Oct 28;13(21):3426. doi: 10.3390/foods13213426 (PMC11545086; doi:10.3390/foods13213426)
Supplement: Supplementary file 1 [file foods-13-03426-s001.zip › foods-3263875-supplementary.pdf]

Supplementary materials

Table S1. Composition and nutrient level of the basic diet

Raw material composition /%	1–21 days of age	22–42 days of age
Corn	58.50	60.10
Soybean oil	3.04	4.50
Soybean meal	28.00	30.00
Fermented soybean meal	5.00	-
Talcum powder	1.60	1.35
Calcium biphosphate	1.40	1.45
Lysine	-	0.10
Methionine	0.14	0.15
Threonine	0.10	0.09
Salt	0.22	0.26
Premix feed[1]	2.00	2.00
Total	100.00	100.00
Nutrient level /%[2]		
Metabolizable energy /MJ·kg^{-1}	12.33	12.76
Crude protein	21.04	18.97
Crude fat	5.60	7.06
Calcium	1.00	0.90
Total phosphorus	0.65	0.60
Available phosphorus	0.47	0.40
Lysine	1.08	1.01
Methionine	0.43	0.40
Methionine + cystine	0.76	0.72
Threonine	0.83	0.77

Note:[1]The premix provides vitamin A 8 500 IU, vitamin D 3 800 IU, vitamin E 25 IU, vitamin K 1.2 mg, vitamin B_1 2.0 mg, vitamin B_2 5.0 mg, vitamin B_6 2.0 mg, niacin 25 mg, pantothenic acid 13.2 mg, biotin 0.18 mg, iodine 0.35 mg, selenium 0.30 mg, manganese 100 mg, iron 80 mg, copper 8 mg, and zinc 75 mg. Premix does not contain any antibiotics and chemical synthetic antibacterial drugs. [2]Metabolizable energy in nutrient levels are calculated values, all others are measured values.

Table S2. Detection parameters on the machine in positive and negative ion mode

Condition	Parameter
Injection volume	2 μL
Sample tray temperature	4°C
Electron Spray Ionization (ESI)	positive (ESI+) and negative (ESI-) ion modes
Flow rates of the sheath gas	30 Arb
Flow rates of the auxiliary gas	25 Arb
Capillary temperature	350°C
Collision energy	10/30/60
Electrospray voltage (ESI+)	3.6 kV
Electrospray voltage (ESI+)	3.2 kV

Table S3. GC-MS online detection conditions

Condition	Parameter
Column type	HP-INNOWax
Injection volume	1 μL
Chromatographic column temperature	An initial temperature of 70°C was maintained for 5 min
Heating speed	Followed by a rise to 200°C at 25°C /min
Injection temperature	250°C
Carrier gas	Helium
Carrier gas flow rate	0.8 mL/min
Split flow ratio	20:1
Ion source temperature	230°C
MS four-stage rod temperature	150°C
Running time	10 min

Table S4. The results of free amino acids composition and content in breast muscle of AA broilers (mg/g)

Group[2] / Free amino acid[1]	CON	CTC	PCP250	PCP500	PCP750	PCP1000
Asp	1.899±0.223	2.509±0.949	2.151±0.468	3.261±0.942	3.385±1.077	1.952±0.171
Glu	5.351±0.757^{b}	5.740±0.325^{b}	6.958±1.806^{b}	5.711±0.249^{b}	9.767±1.797^{a}	7.064±0.883^{b}
Gly	1.396±0.229	2.010±0.814	1.607±0.333	2.359±0.653	1.738±0.392	2.424±0.703
Ala	1.773±0.360^{b}	2.920±1.219^{a}	2.246±0.440^{ab}	2.951±0.545^{a}	2.958±0.165^{a}	2.112±0.211^{ab}
Thr	1.659±0.240	2.060±0.686	1.694±0.323	2.128±0.346	2.165±0.411	2.621±0.719
Ser	1.245±0.148	1.545±0.723	1.418±0.310	1.933±0.377	2.098±0.255	1.899±0.548
Val	1.699±0.210	2.321±0.956	1.893±0.386	2.334±0.364	2.987±0.928	1.798±0.088
Met	0.465±0.076^{c}	0.872±0.443^{bc}	0.869±0.392^{bc}	1.266±0.209^{abc}	2.171±0.343^{a}	1.493±0.782^{ab}
Ile	1.610±0.181	2.009±0.663	1.707±0.326	2.199±0.366	2.541±0.965	2.672±0.801
Leu	2.512±0.260^{b}	3.124±0.991^{ab}	2.829±0.585^{ab}	3.559±0.536^{ab}	2.893±0.274^{ab}	4.384±1.390^{a}
Tyr	1.127±0.161^{b}	1.589±0.721^{ab}	1.246±0.282^{b}	1.909±0.536^{a}	1.804±0.370^{a}	1.984±0.650^{a}
Phe	3.093±0.197^{b}	3.048±1.601^{b}	4.802±0.942^{b}	4.109±0.511^{b}	4.126±0.382^{b}	6.965±0.608^{a}
Arg	0.791±0.488^{b}	1.457±0.187^{a}	2.076±0.728^{a}	1.869±0.373^{a}	2.417±0.315^{a}	2.168±0.437^{a}
His	0.865±0.492	0.433±0.508	0.378±0.494	0.044±0.011	0.024±0.003	0.421±0.518
Lys	2.426±1.003	1.805±1.158	1.353±1.211	0.422±0.216	0.505±0.161	1.486±1.139
Pro	0.000±0.000^{d}	1.271±0.251^{a}	0.290±0.120^{cd}	0.409±0.112^{bc}	0.402±0.153^{bc}	0.153±0.681^{b}
Orn	1.994±0.048^{cd}	1.763±0.000^{d}	3.036±0.450^{b}	2.979±0.530^{b}	2.628±0.253^{bc}	3.977±0.363^{a}
Cys	0.303±0.054^{ab}	0.349±0.111^{a}	0.173±0.143^{ab}	0.095±0.042^{b}	0.083±0.023^{b}	0.171±0.118^{ab}
Umami AAs	7.250±0.885^{b}	8.249±0.819^{b}	9.109±2.122^{b}	8.972±1.151^{b}	13.151±2.873^{a}	9.016±0.712^{b}
Sweet AAs	6.073±0.663	8.535±3.421	6.965±1.398	9.371±1.882	8.958±0.344	9.055±1.892
Bitter AAs	12.162±1.030^{c}	14.854±4.937^{bc}	15.798±3.276^{abc}	17.288±1.024^{abc}	18.962±1.267^{ab}	21.884±3.534^{a}
Tasteless AAs	2.426±1.003	3.076±1.161	1.643±1.151	0.832±0.326	0.907±0.258	2.168±1.126
Others	2.297±0.082^{c}	2.113±0.111^{c}	3.209±0.474^{b}	3.074±0.506^{b}	2.711±0.240^{bc}	4.147±0.251^{a}
Total AAs	30.207±2.268^{b}	36.826±9.344^{ab}	36.726±6.790^{ab}	39.537±4.024^{ab}	44.689±3.743^{a}	46.270±4.531^{a}

Mean values with different superscript letters within the same row are significantly different ($P < 0.05$). The results are represented as mean values with SEM (n = 6).

[1]Umami AAs = sum of (Asp, Glu); Sweet AAs = sum of (Gly, Ala, Thr, Ser); Bitter AAs = sum of (Val, Met, Ile, Leu, Tyr, Phe, Arg, His); Tasteless AAs = sum of (Lys, Pro, Orn, Cys).

[2]CON represents the blank group, CTC represents the antibiotic group, and PCP represents different doses of *Pleurotus Citrinopileatus* polysaccharide treatment groups

Table S5. Metabolites in breast muscle of AA broilers in positive ion mode

ID	Metabolites	ID	Metabolites	ID	Metabolites
1	Hypoxanthine	51	Guanosine	141	Glycerol 3-phosphate
2	Betaine	54	Phosphorylcholine	142	O-Propanoyl-carnitine
3	Adenine	56	SPhingosine	143	Histidinyl-Aspartate
4	L-Proline	57	Oleamide	151	Homoanserine
5	L-alpha-Aminobutyric acid	60	L-Glutamic acid	152	Methoxypyrazine
6	Allopurinol riboside	62	Argininic acid	158	Linamarin
7	Zymonic acid	63	Butyrylcarnitine	160	Citicoline
8	Cytosine	64	Adenosine 2'-phosphate	161	Ethyl glucuronide
9	Choline	65	D-1-Piperideine-2-carboxylic acid	163	Pinostilbenoside
10	D-Alanine	66	Arginyl-Valine	175	Palmidin A
11	Deoxyguanosine	72	Dihydroxybenzoyloxy)-4,6-dihydroxybenzoate	178	L-Glutamine
12	Creatine	74	N-Acetylglutamine	190	Phosphocreatine
13	L-Carnitine	75	5-Hydroxy-L-tryptophan	193	Homocysteine thiolactone
15	L-Histidine	79	2-Methylbutyroylcarnitine	200	4-Hydroxy-1-(3-pyridinyl)-1-butanone
16	Creatinine	83	2-O-(5,8,11,14,17-Eicosapentaenoyl)-1-O-hexadecylglycero-3-phosphocholine	205	Homoarecoline
18	Proline betaine	86	D-Alanyl-D-alanine	206	Cytarabine
19	IMP	89	Phosphocholine	211	Pantothenic acid
20	Niacinamide	94	Prolyl-Valine	212	3-(5-Methyl-2-furanyl)butanal
21	N-Acetylhistidine	103	Homo-L-arginine	227	4-(Methylnitrosamino)-1-(1-oxido-3-pyridinyl)-1-butanone
22	4-Guanidinobutanoic acid	104	ADP	246	Histidinyl-Proline
23	L-Phenylalanine	108	Imidazole-4-acetaldehyde	266	Isopentyl mercaptan
28	P-Aminobenzoic acid	111	NAD	271	5-Acetylamino-6-formylamino-3-methyluracil
29	beta-Alanine	113	Dimethyl fumarate	277	Isoleucyl-Alanine
33	Dopamine quinone	114	Formiminoglutamic acid		
34	N-Nitroso-pyrrolidine	115	Methylimidazoleacetic acid		
35	Pyroglutamic acid	116	3-Amino-2-piperidone		
36	5'-Methylthioadenosine	118	2-Keto-6-acetamidocaproate		
39	3-Methylhistidine	119	Glutamine		
40	Uridine	121	Prolyl-Alanine		
43	Anserine	122	Aspartyl-Histidine		
44	3-Indoleacetonitrile	124	Arginyl-Alanine		
45	Taurine	126	Acetone cyanohydrin		
47	Glycine	128	Nebularine		
48	Ascorbyl stearate	130	Hydroxyprolyl-Histidine		
49	L-Arginine	134	L-Leucine		
50	Cysteinyl-Tyrosine	139	Isotheaflavin		

Table S6. Metabolites in breast muscle of broilers in negative ion mode

ID	Metabolites	ID	Metabolites	ID	Metabolites
2	L-Norleucine	53	Citric acid	82	Phenyllactic acid
3	Oleic acid	54	Caprylic acid	83	N-Acetyl-L-Phenylalanine
4	Palmitic acid	55	Isokobusone	84	4-Hydroxy tolbutamide
5	Pyruvic acid	56	3-Methyl-2-oxovaleric acid	86	Anserine
6	Dodecanoic acid	57	Terephthalic acid	87	Inosine
8	Palmitoleic acid	58	Pyroglutamic acid	88	Glucose 6-phosphate
9	Isopalmitic acid	59	2-Hydroxycinnamic acid	91	Sorbitol
10	Eicosadienoic acid	60	Hexadecanedioic acid	92	Succinate Phosphocholine
11	Phosphocreatine	62	Uridine	93	4-Pyridoxic acid
12	Adrenic acid	63	Imidazoleacetic acid	96	Uridine 5'-monophosphate
13	D-Tagatose	64	L-Serine	97	L-Asparagine
14	L-Proline	66	Glutaric acid	99	L-Gulonolactone
15	Pelargonic acid	67	Maleic acid	101	IMP
17	Tauroursodeoxycholic acid	68	D-Malic acid	102	Succinic acid semialdehyde
18	Fructose 6-phosphate	56	3-Methyl-2-oxovaleric acid	103	myo-Inositol
19	Adenine	57	Terephthalic acid	104	Hydrogen phosphate
22	Uracil	58	Pyroglutamic acid	106	N-Acetylaspartylglutamic acid
24	Arachidonic acid	59	2-Hydroxycinnamic acid	107	Pseudouridine
25	Phosphoenolpyruvic acid	60	Hexadecanedioic acid	108	Acetylglycine
26	L-Malic acid	62	Uridine	109	Pantothenol
27	3-Methylhistidine	63	Imidazoleacetic acid	112	Ribothymidine
28	Pyrrole-2-carboxylic acid	64	L-Serine	113	N-Acetylleucine
30	Creatinine	66	Glutaric acid	115	Mesaconic acid
32	Undecanoic acid	67	Maleic acid	116	Beta-Guanidinopropionic acid
37	L-Glutamic acid	68	D-Malic acid	118	O-Phosphoethanolamine
38	4-Dodecylbenzenesulfonic Acid	70	5'-Methylthioadenosine	119	Threonic acid
39	Malic acid	71	D-Xylitol	120	cis-Aconitic acid
41	Phenylpyruvic acid	72	Histamine	121	N-Formyl-L-aspartate
43	Phthalic acid	73	Adenosine	122	Ethyl hexadecanoate
44	Ethyl dodecanoate	74	Valeric acid	123	N-Acetyl-D-Glucosamine 6-Phosphate
45	D-Glyceraldehyde 3-phosphate	75	D-Glutamine	124	Capric acid
46	Ethyl oleate	76	Citraconic acid	125	Deoxyuridine
47	(R)-3-Hydroxy-tetradecanoic acid	77	Carnosine	126	Uridine diphosphategalactose
48	Chenodeoxycholic acid	78	3-Hydroxymethylglutaric acid	128	Taurine
49	Benzoic acid	79	Cytidine monophosphate	130	Citramalic acid
50	Pyrophosphate	80	Gamma-Linolenic acid	131	L-Valine
52	L-Histidine	81	Gingerol	132	S-Adenosylhomocysteine

ID	Metabolites	ID	Metabolites	ID	Metabolites
133	Thymidine	154	D-Ribose	170	Itaconic acid
135	Deoxyinosine	155	3-Methoxy-4-hydroxyphenylethyleneglycol sulfate	171	D-Alanyl-D-alanine
136	N-Acetylserine	156	dUMP	172	Cytidine
137	Theaflavin	157	5-Hydroxy-L-tryptophan	174	Guanosine monophosphate
138	N-Acetylglutamic acid	160	Fexofenadine	176	Parabanic Acid
139	Guanosine	162	Fructose 1,6-bisphosphate	178	Dethiobiotin
141	Malonic acid	163	Galacturonic acid	181	D-Alanine
142	Gluconolactone	164	NAD	182	8-Hydroxyguanosine
143	1-deoxy-1-(N6-lysino)-D-fructose	165	Allantoin	183	cis-Vaccenic acid
144	Threonine	166	N4-Acetylcytidine	186	5-Methylcytidine
149	N-Acetylhistidine	167	dGTP	189	Acetaminophen
150	Aminoadipic acid	168	2-Oxovaleric acid	190	N-Acetyl-L-alanine
153	7,8-Dihydroneopterin	169	D-Glycero-D-galacto-heptitol		

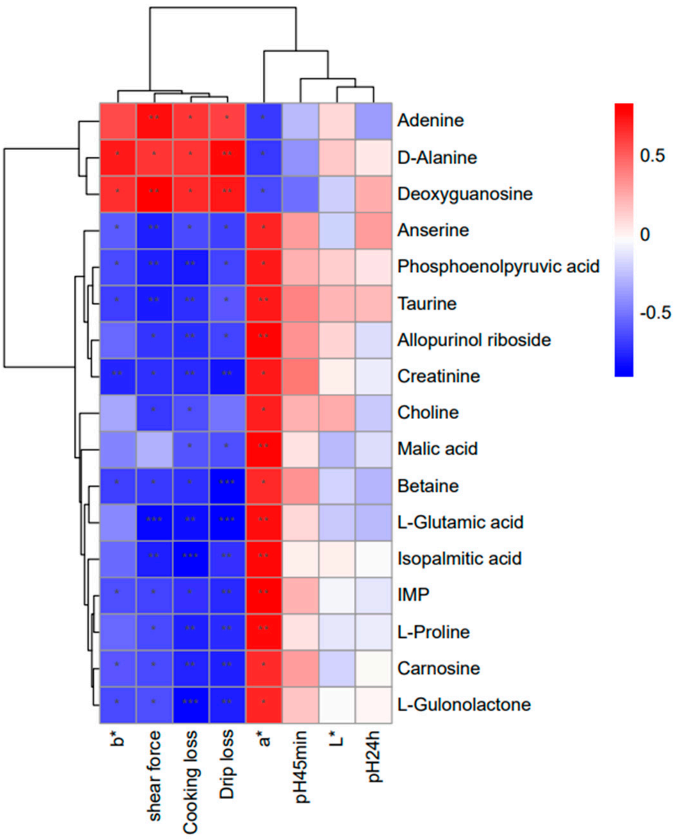

Figure S1. Correlations between key meat parameters and differential metabolites of AA broilers. Significant correlation is marked by **P*<0.05, ***P*<0.01, ****P*<0.001.

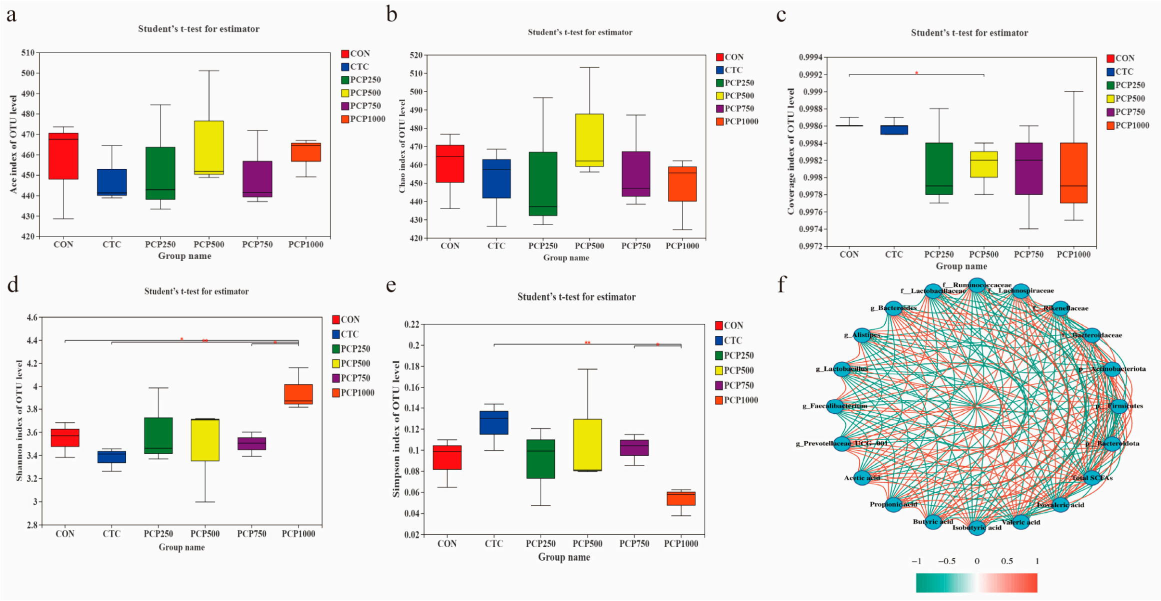

Figure S2. Analysis chart of influence of caecal alpha diversity in each group. (a) ace index; (b) Chao index; (c) coverage index; (d) Shannon index; (e) Simpson index. Significant correlation is marked by *P<0.05, **P<0.01. (f) Correlation network analysis of microorganisms and short-chain fatty acids.
